# Cystic form of Actinomycotic mycetoma: A new case with a diagnostic challenge

**DOI:** 10.1002/ccr3.4064

**Published:** 2021-03-16

**Authors:** Ahlem Bellalah, Nouha Ben Abdeljelil, Manel Njima, Seifeddine Ben Hammouda, Sarah Ben Khalifa, Mustapha Koubaa, Abdelfatteh Zakhama, Rim Hadhri

**Affiliations:** ^1^ Department of Pathology Fattouma Bourguiba University Hospital Monastir Tunisia; ^2^ Faculty of Medicine University of Monastir Monastir Tunisia; ^3^ Department of Orthopedic Surgery Fattouma Bourguiba University Hospital Monastir Tunisia

**Keywords:** actinomycosis, cyst, histopathology, mycetoma

## Abstract

Mycetoma, commonly known as Madura foot, is a chronic granulomatous infection caused either by fungi (eumycetoma) known as actinomycete. This disease occurs preferentially in young adults, and it affects the foot in particular. We report a Tunisian case of mycetoma occurring in an old patient, particular by its cystic presentation.

## Graphical abstract

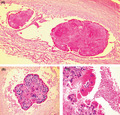

## 1 INTRODUCTION

Mycetoma, commonly known as Madura foot, is a localized, chronic granulomatous infection that is caused either by fungi (eumycetoma) or by aerobic filamentous bacteria known as actinomycete (actinomycetoma). It affects usually extremities involving deep dermis and subcutaneous tissue, where it is inoculated by minor trauma.

We report a new Tunisian case of mycetoma particular by its cystic presentation and by the absence of discharge.

## 2 CASE REPORT

**FIGURE 1:**
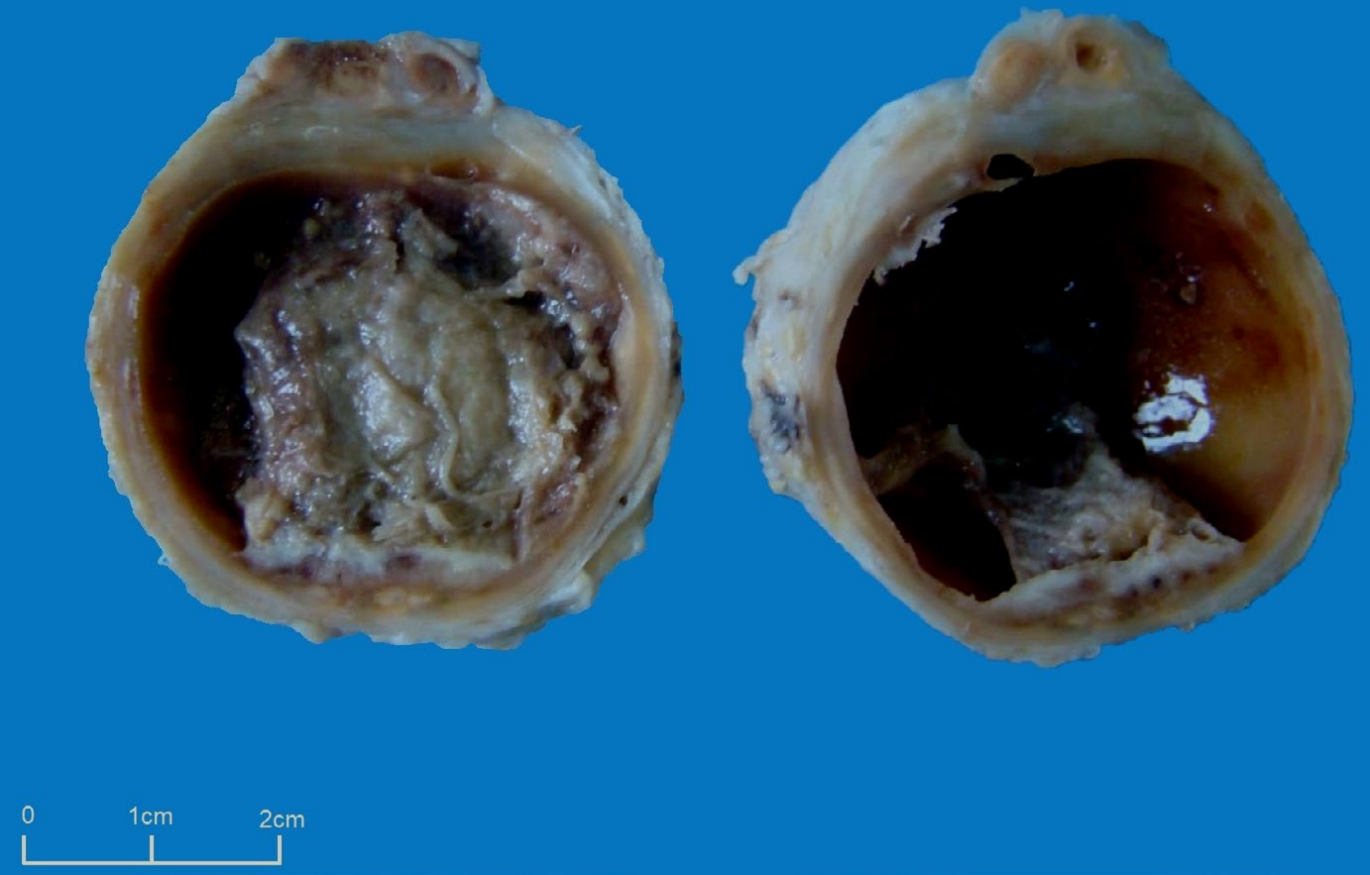
Gross examination showing a cystic mass surrounded by a thick capsule with a crumbly white fluid

A 74‐year‐old man presented to the emergency room of our hospital, with a 10‐year history of progressive painless swelling of the left foot. This patient, otherwise healthy, belonged to rural backgrounds and worked in farming. He specifically denied any knowledge of injury to his foot. Physical examination found a tumor‐like lesion on the dorsal surface of his left foot, which was painless and firm in consistency. The overlying skin displayed no erythema, cutaneous changes, or draining sinuses, and its temperature was normal. There was no regional lymphadenopathy. Ultrasonic examination revealed a multilocular cystic mass with a thick capsule and heterogeneous fluid suggestive of synovial cyst. The patient underwent wide local excision without bacteriologic test. The excised mass measured 4.5 × 4.5 × 3 cm. On cut surface, it consisted in a cystic formation surrounded by a thick fibrous capsule and contained a crumbly white fluid (Figure 1).

**FIGURE 2:**
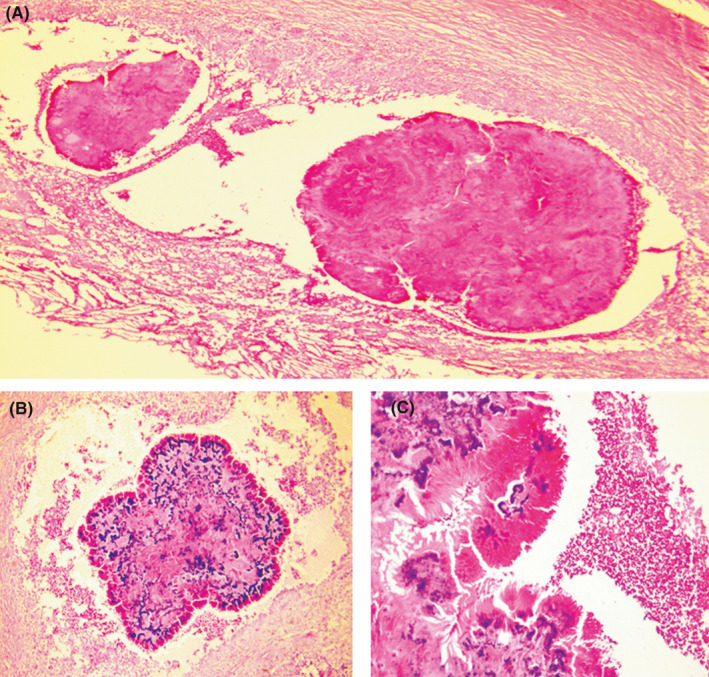
Mycetoma. A, Histological findings showing a cyst with a fibrous capsule and a content consisting in a chronic granulomatous inflammation (HE ×40). B, Medium power‐view with an acute inflammatory reaction surrounding several basophile grains, strongly suggestive of actinomycotic mycetoma (HE ×200). C. High power‐view showing longitudinal hyphae at the periphery the actinomycotic grain (HE ×400)

Histologically, the capsule was made of a thick fibrous tissue with a foreign body reaction around lipid crystals (Figure 2A). The content of this cyst consisted in a chronic granulomatous inflammation with a central focus of acute inflammatory reaction surrounding several basophile grains (Figure 2B). The granule showed a branching filaments arranged in a radial pattern strongly suggestive of actinomycotic mycetoma (Figure 2C). This granule was positive with the Gram stain and negative with Ziehl neelsen and periodic acid–Schiff stains. The diagnosis of actinomycotic mycetoma was established.

The patient received an antibiotherapy (streptomycin and co‐trimoxazole) with favorable outcome.

## 3 DISCUSSION

Mycetoma is a chronic progressive granulomatous inflammation of subcutaneous tissue, skin, and bones recognized by the World Health Organization as a “neglected tropical disease”.^1^ It was first described in Madurai in India by Gill in 1842 and was initially called Madura foot.^2^ These infections may be caused by fungi and termed eumycotic mycetoma or eumycetoma, or by gram‐positive aerobic filamentous bacteria, termed actinomycotic mycetoma or actinomycetoma.^3^ Mycetoma can be caused by a number of organisms.

Common actinomycotic species include Actinomadura madurae, Streptomyces somaliensis, Nocardia brasiliensis, Actinomadura pelletieri, and Nocardia asteroids,^4^ and common eumycotic agents are Madurella mycetomatis, Madurella grisea, Pseudoallescheria boydii, and Leptosphaeria senegalensis.^4^ This disease is most commonly found in tropical and subtropical climates in India, Sudan, Somalia, Senegal, Yemen, Mexico, and Venezuela.^3^, ^4^, ^5^, ^6^ Mycetoma in Tunisia is uncommon and only observed sporadically with a slight female predominance. The actinomycotic agents in particular Actinomadura madurae seem to be the most incriminated in Tunisian cases.^7^, ^8^

This disease occurs preferentially in rural areas, usually among laborers who work barefoot. Therefore, it is more common in males than females and it affects the foot in particular (70% of cases) and hand (15%).^2^, ^4^ Unlike our case which occurred in an old patient aged of 74 years, this condition is common in young adults (16‐40 years old).^3^, ^9^

Clinical suspicion of mycetoma is based on the triad that includes progressive painless subcutaneous swelling, sinus tract formation, and granular discharge.^10^

Our patient presented with a subcutaneous lump on the dorsum of his foot, not associated with sinuses or discharge as in more advanced cases.

Radiological studies can help to define the extent of disease and the involvement of bone. Magnetic resonance imaging may be useful for the early diagnosis of mycetoma with dot‐in‐circle appearance, high‐intensity lesion on T2 images with a tiny central low‐signal focus representing fungal grains within inflammatory granuloma.^11^

Our case is rather particular as regards the age of our patient, the absence of tracts and granular discharge, and the cystic form of mycetoma that lead initially to the diagnosis of synovial cyst. Cystic presentation of mycetoma is exceptional. In the study of Bonifaz, only two cases between 482 have a cystic presentation (0.41%), whereas 97% of them presented as tumor‐like with draining sinuses.^3^, ^12^ Fahal has reported 4 cases of cystic mycetoma caused by Madurella mycetomatis unassociated with overlying sinuses making the diagnosis of mycetoma less obvious. The character of the fluid within the cyst was an exudate due to leakage from the granulation tissue lining the thick capsules. Fahal explained that this exudate may be absorbed by nearby blood vessels and lymphatics in usual form of mycetoma, while it is accumulated in cystic form due to encapsulation.^13^

In endemic countries, all subcutaneous masses are considered as mycetoma until proven otherwise. In other situations, mycetoma can mimic tuberculosis, osteomyelitis, other fungal infections, and tumors of soft tissues.^14^

It is important to recognize the causative agent of mycetoma in order to choose the appropriate treatment. The color of the grains in the discharge from the sinuses can be helpful for presumptive identification of the germ. However, it is not entirely reliable, and recovery of the causative agents in culture is more accurate.

Fine needle aspiration can be used with a sensitivity and specificity of 88.7% for the diagnosis of Madurella mycetomatis.^15^

The key to diagnosis is histological examination. It reveals suppurative granuloma with mycetoma grains. Grains may not be seen on histopathologic section, but when present, its large size and surrounding cluster of neutrophils make it difficult to miss, even without fungal or bacterial stains. Mycetoma grains are usually 0.2 to 5 mm in diameter and thus may be observed grossly without magnification. Histological distinction between fungi and actinomycetes is possible: Madurella mycetomis grains tend to be large, light to dark brown in color, irregular outlines and tend to be fracture the former are recognized by the production of hyphae, whereas the latter are noticed by the presence of filaments (16]. Periodic acid–Schiff (PAS), Gomori methenamine silver, and gram stain along with morphology of grains are helpful in differentiating organisms histopathologically.^16^, ^17^

Culture of grains can be used also to diagnose the specific agent of mycetoma: Specimens should be cultured on mycologic and mycobacteriologic media. Colonies of actinomycetes grow after 7 to 10 days of incubation.^16^ However, delayed diagnosis can lead to severe consequences including amputation. Therefore, histopathologic examination is useful for the early diagnosis of mycetoma.

Molecular diagnosis of the causative agent by direct sequencing of biopsy specimens can provide a rapid diagnosis using 16s RNA gene sequencing studies for actinomycetes and pan‐fungal PCR for eumycetes.^18^, ^19^

Treatment of this disease is often challenging and depends on the microbiological findings (bacterial or fungal) and the extent of the lesions. Typically, it includes antimicrobial agents and surgery.^20^

Unlike eumycetomas where antifungal therapy based on azole antifungals should be continued for 2‐4 years, medical treatment of actinomycetoma is generally more efficient. The most commonly described regimens for actinomycetoma include streptomycin associated with either co‐trimoxazole or dapsone. Combination therapy of two or more drugs is often used to prevent resistance to one antibiotic and persistence of the infection. Duration of treatment varies overall from 3 to 24 months. Surgical excision is indicated in localized lesions, resistance to medical treatment. Amputation is reserved to very advanced diseases.^20^

## 4 CONCLUSION

Although mycetoma is a rare lesion outside tropical countries, it should be diagnosed early to begin treatment and avoid complications. A delay of 4 weeks is necessary to obtain culture results, hence the importance of histological findings.

## ETHICAL CONSIDERATIONS

The patient was informed in detail, and he provided written consent.

## CONFLICT OF INTEREST

None declared.

## AUTHOR CONTRIBUTIONS

Ahlem Bellalah, Nouha Ben Abdeljelil, Seifeddine Ben Hammouda, and Sarah Ben Khalifa: prepared the manuscript. Manel Njima, Mustapha Koubaa, Abdelfatteh Zakhama, and Rim Hadhri: guided authors in writing the manuscript and proofread the final manuscript.

## Data Availability

All data relevant to the study are included in the article or uploaded as supplementary information.
